# Wave Dissipation and Energy-Absorption Characteristics of Wave-Absorbing Metal Plates with Different Aperture Sizes and Thicknesses under True-Triaxial Static-Dynamic-Coupling Loading

**DOI:** 10.3390/ma15103493

**Published:** 2022-05-12

**Authors:** Linqi Huang, Xin Wu, Sijian Zeng, Xibing Li

**Affiliations:** School of Resources and Safety Engineering, Central South University, Changsha 410083, China; huanglinqi@csu.edu.cn (L.H.); csu_wuxin@csu.edu.cn (X.W.); sjzcsu@163.com (S.Z.)

**Keywords:** SHPB, wave-absorbing metal plates, static-dynamic-coupling loading, energy consumption law, boundary materials, stress waves, wave energy absorption

## Abstract

Deep rock masses exist in a complex environment with multi-field coupling; therefore, it is necessary to develop a true-triaxial static-dynamic-coupling loading test machine to explore their characteristics and mechanical response mechanism. To meet the test requirements of true-triaxial loading and strong disturbance, a wave-absorbing metal plate was selected as the boundary material between the granite and transmission end, and the modified SHPB was used to perform static-dynamic-coupling loading tests. In this study, two series of experiments on wave- absorbing metal plates were conducted, which were fixed aperture sizes with different thicknesses and fixed thicknesses with different aperture sizes. The static-dynamic-coupling loading tests on each aperture size and plate thickness were carried out under the condition of equal energy impact. The effects of the aperture size and plate thickness on the incident- and reflection-stress curves, reflectivity, energy consumption law, energy evolution, and other mechanical properties of the wave-absorbing metal plate materials were studied. The results show that the peak stress and reflectivity decrease with increasing aperture size and plate thickness, and the influence of the thickness is greater than that of the aperture size. The energy-absorption rate of the wave-absorbing metal plate increased with increasing thickness and aperture size and was maximized when the aperture size and thickness were 6–7 mm and 3–4 mm, respectively. The variation trend of the energy reflectance is opposite to that of the energy absorption and reaches a minimum when the aperture size is 6–7 mm and plate thickness is 3–4 mm. The energy transmittance of the wave-absorbing metal plate fluctuated in a stable range, but the variation range was less obvious compared to that of the energy-absorption rate.

## 1. Introduction

**Figure 1:**
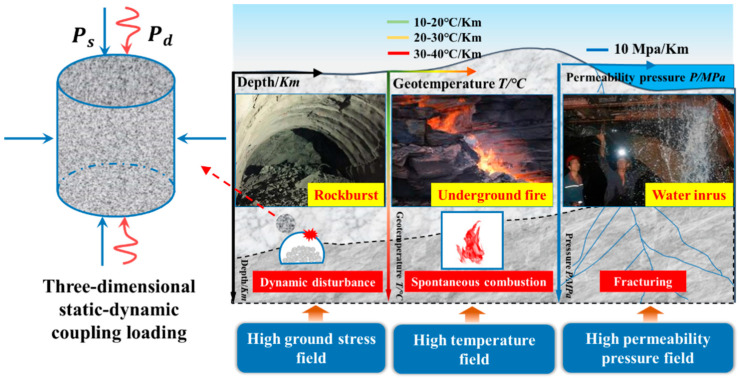
Deep-rock-mass “three-high” multi-field coupling environment.

With the continuous development of shallow resources and the increasing demand for human mineral resources, delving deep into the earth has become a strategic problem [1,2,3,4]. The depth of the Mponeng mine in South Africa, the deepest mine in the world, has surpassed 4000 m [5] and as a result of the development of mineral resources in China, mines in the country will reach an underground depth of 1000–2000 m [6,7]. Deep rock mass occurs in a complex environment consisting of a high-stress field, high-temperature field, high permeability–pressure field, fast unloading, and dynamic disturbance caused by hard-rock mining, resulting in frequent disasters [8,9,10,11,12], as shown in Figure 1. For example, deep mining of the Kaiyang phosphate mine has led to a wide range of loose circles in the rock surrounding the underground engineering owing to the action of high stress; this simultaneously results in decreased rock strength, increased unstable block, and groundwater seepage, which has caused great engineering geological disasters [13,14,15]. For example, plate cracks and rock bursts occurred in the side wall of a 500 m underground tunnel at the west mountain entrance of the Linglong gold mine in Shandong Province [16]. On 21 September 2001, a strong rockburst occurred at a distance of 9 + 122 m, and a damage pit of 1.8 m was formed at the Jinping II hydropower station [17]. These deep-rock-mass disasters are related to the specific stress environment and engineering disturbances of the surrounding deep rocks [18]. In the laboratory, we usually use a true-triaxial static-dynamic-coupling loading system to conduct reproducible scientific tests on such field disasters [19,20]. However, owing to the thinness of the loading plate in the conventional true-triaxial pressure chamber and the inconsistent wave-impedance matching between the loading plate and rock sample, the stress wave is reflected when it reaches the interface between the loading plate and sample, which seriously affects the test parameters. In addition, such boundary conditions significantly affect the accuracy of true-triaxial loading tests. Therefore, it is very important to select an appropriate boundary material to be placed between the rock and transmission end to eliminate the transmitted wave, reflected wave, and absorbed energy in order to meet the test requirements under true-triaxial strong disturbance and to improve the accuracy of true triaxial static-dynamic-coupling loading tests.

The energy evolution law and stress-wave energy-absorption characteristics of the boundary materials in test systems have been previously studied. Shi et al. [21] developed a set of model test systems for catastrophic evolution and instability of deep-roadway rock bursts and added a special aluminum-alloy wave-suppression plate with a 5 mm hole to the front end of the model-load plate of the test system. The aluminum-alloy wave-suppression plate attenuates the reflected wave generated when the explosion shock reaches the boundary of the model and overcomes the drawback, in which the repeated action of the explosion-stress wave in the simulated tunnel does not match the scene. Dong et al. [22] conducted an experimental study on stress-wave propagation in multilayered media containing foamed concrete and pointed out that for combined media with a large degree of wave-impedance mismatch, multiple reflections and transmissions between adjacent media were required before equilibrium was reached. This means that the more mismatched the media, the more reflection and transmission occur in the multilayered media. Tang et al. [23] studied the action mechanism of the energy-dissipating layer of foamed concrete and found that appropriately selecting the properties and thickness of the energy-dissipating layer material can reduce the pressure acting on the structure and improve its uneven distribution. In the model test device of anti-explosion structure in geotechnical engineering, the explosion-reflected wave would have a great influence on the experimental results. In order to reduce this influence, Shen et al. [24] set an aluminum-alloy wave-suppression plate with a certain thickness on the inner wall of an anti-explosion box and on the bottom of a foundation pit; this strategy successfully led to wave suppression. Zhang et al. [25] found that aluminum-foam-sandwich boards have good wave dissipation and energy-absorption effects on air and in underwater explosions when comparing and analyzing the propagation and attenuation law of shock explosion waves in air and water. Based on impedance-matching theory, Li et al. [26,27] proposed a solution for cases in which the impedance of the explosion and rock mass is very mismatched: if an intermediate layer with appropriate impedance and thickness is used, the energy utilization rate of the explosive can be improved. Feng et al. [28] used MTS(Mechanical Testing and Simulation) and SHPB(Split Hopkinson Pressure Bar) to study the influence of strain rate on the compressive mechanical properties and energy-absorption properties of foam-based aluminum alloys in the strain-rate range of 10 s^−3^ to 2600 s^−1^. Li et al. [29] pointed out that when the stress wave passed through the foam material, the momentum and energy decreased, but the amplitude of the stress wave increased. Guruprasad et al. [30,31] proposed a new type of energy-absorption device and analyzed and discussed its energy-absorption characteristics under blast loads through both experiments and numerical simulations. Han et al. [32] used an improved separated Hopkinson-pressure-bar system to conduct dynamic impact tests on sandstone specimens sandwich-filled with cement mortar of different thicknesses. They found that the transmission coefficient, dynamic strength, and energy absorption decreased with an increase in joint thickness, but the reflection coefficient, peak strain, and joint closure showed opposite trends. Beggs et al. [33] conducted dynamic tests on AZ31B magnesium alloy samples with four cylindrical shapes using a compressed split Hopkinson bar, and their results indicated that the energy-absorption performance of the sample improved significantly with an increase in strain rate. Nammi et al. [34] used the finite element method to study the effect of bubble size on the compression response and energy-absorption characteristics of closed-cell aluminum foam. The results showed that a small pore size led to a larger specific surface area and volume ratio as well as higher peak stress and compact strain. These results reveal that the energy-absorption characteristics of the boundary materials are related to their aperture and thickness.

However, the comprehensive performance of energy absorption and wave elimination of boundary materials under true-triaxial and strong-disturbance conditions is rarely required in real-world experimental studies. Since the compression and tensile waves generated by the boundary material cancel each other near the boundary, wave elimination can be achieved. Therefore, the study of the energy-absorption characteristics of different boundary materials under static-dynamic-coupling loading has guiding significance for the development of true-triaxial static-dynamic coupling-loading test instruments. The results show that the attenuation effect of the wave-absorbing metal plate on the reflected stress is more prominent than that of the porous material when the wave-absorbing energy characteristics of different materials are impacted by equal energy in a one-dimensional (1D) static-dynamic coupling-loading test system, and the wave-absorbing metal plate has better repeatability and fatigue resistance [35,36,37,38]. On this basis, the current study uses a wave-absorbing metal plate as the research object and an SHPB device to study the wave-absorbing energy characteristics and energy-evolution law of wave-absorbing metal plates with different aperture sizes and thicknesses under static-dynamic coupling-loading conditions.

## 2. Experimental Programs

### 2.1. Experiment Material

**Figure 2:**
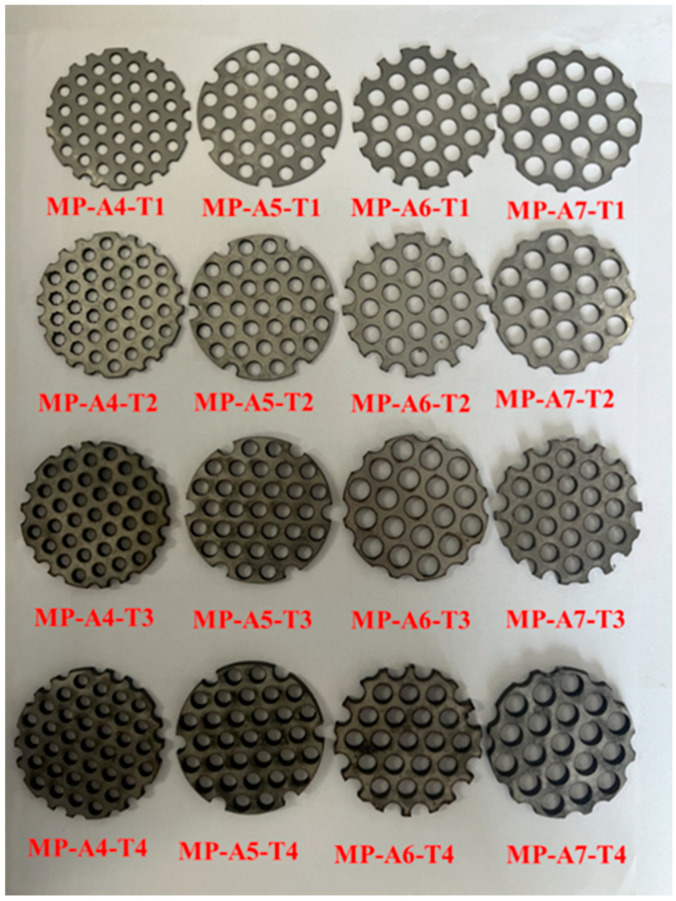
Types of wave-absorbing metal plates.

MP (Mechanical polishing) refers to polishing the surface of a stainless steel tube with a polishing wheel or polishing belt, achieving a smooth polishing effect. MP304 stainless steel material contains carbon, silicon, manganese, phosphorus, sulfur, nickel, chromium and other components, and the test shows that its yield strength is greater than 205 Mpa, and the tensile strength is greater than 520 Mpa [39,40]. Wave-absorbing metal plate refers to making different shapes of holes in metal materials to meet different needs. It is widely used in the electronic industry, chemical machinery, noise-absorbing equipment and anti-detonation device [41]. In this study, a circular-hole wave-absorbing metal plate with a 60-degree arrangement of the MP304 stainless steel was selected. This research shows that the wave-absorbing metal plate performs well in all aspects. Therefore, considering the plate as the research object and the aperture size and plate thickness to be influencing factors, four series of apertures and thicknesses were selected, as shown in Figure 2. The wave velocity of the wave-absorbing metal plate, the boundary test material selected for the sample, was 1470 m/s, the density was 7930 $\mathrm{kg}/m^{3}$, and the wave impedance was 11,657 $\left(g/{\mathrm{cm}}^{3}\right)\cdot \left(m/s\right)$. Table 1 lists the geometrical dimensions of the selected test specimens, where the sample number represents four aperture sizes and four thicknesses of the wave- absorbing metal plate. For example, MP-A4-T1 represents a wave-absorbing metal-plate sample with an aperture size of 4 mm and plate thickness of 1 mm.

### 2.2. Experimental Setup

**Figure 3:**
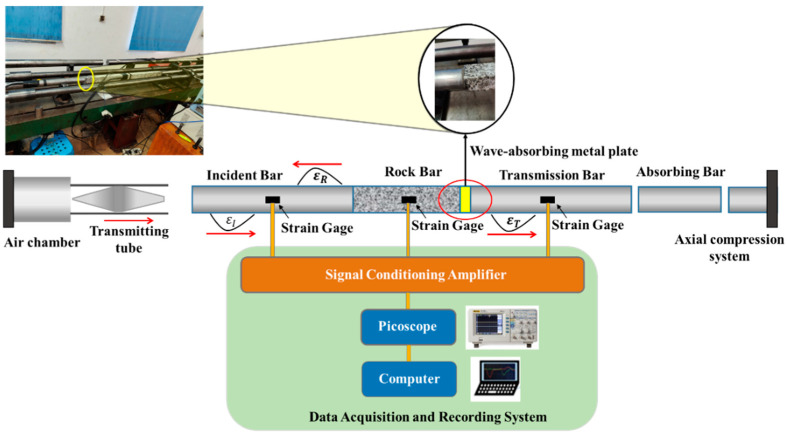
Schematic diagram of SHPB test system under static-dynamic-coupling loading.

This study was conducted using a rock combined-dynamic-and-static loading-test system based on the SHPB device developed by Central South University [1,42,43]. Figure 3 shows a schematic of the test setup, which consists of an incident bar, transmission bar, absorption bar, spindle punch, and data acquisition system. The data acquisition system includes a strain gauge, ultra-dynamic strain gauge, and picoscope combined with a computer. Under the combined action of the true-triaxial pressure test and strong-disturbance loading, the test parameters were affected by factors such as the mismatch of wave impedance at both ends of the rock. Therefore, in this study, changes were made to the SHPB hybrid loading device developed at Central South University in order to explore the wave-absorbing characteristics of boundary test materials with different aperture sizes and thicknesses.

As shown in Figure 3, a long, square granite bar was inserted between the incident and transmission bars. During the experiment, the boundary test material was placed between the granite bar and transmission bar. Considering the wavelength of the spindle punch, and to ensure that the incident and reflected waves received by the strain gauge of the granite bar did not overlap, the length of the granite bar was set to 1 m. Strain gauges were mounted in the middle of the incident bar, rock bar, and transmission bar, and the corresponding oscilloscope, ultra-dynamic strain gauge, and other signal-acquisition equipment were equipped. The test equipment parameters are listed in Table 2. To avoid one-time impact damage and achieve the effect of cyclic impact, under the condition that the position of the bullet in the firing chamber remains unchanged, an appropriate impact pressure value was selected to carry out experimental impact loading on the granite bar. When the cyclic impact test was performed under each axial pressure series, the position of the fixed punch in the launching chamber remained unchanged, and the cyclic impact test was performed at a pressure lower than the critical pressure value until the sample was destroyed. In the cyclic impact process, if the same pressure value is used for each test, it has an equal-amplitude cyclic impact.

## 3. Analysis and Discussion

### 3.1. Energy Consumption

This study employed a separated-Hopkinson-bar test device that gathers data on the mechanical properties of materials under a high strain rate. The fundamental principle is based on the 1D stress-wave-propagation theory, and the changes in the stress, strain, and strain rate of specimens over time are solved by the transmitted and reflected waves on both sides of the specimen [1,41,42,43]:

(1) $$\sigma \left(t\right)=\left[\sigma_{I}\left(t\right)-\sigma_{R}\left(t\right)+\sigma_{T}\left(t\right)\right]A_{e}/\left(2A_{s}\right)$$

(2) $$\varepsilon \left(t\right)=\frac{1}{\rho_{e}C_{e}L_{s}}\int_{0}^{t}\left[\sigma_{I}\left(t\right)+\sigma_{R}\left(t\right)-\sigma_{T}\left(t\right)\right]dt$$

(3) $$\dot{\varepsilon }\left(t\right)=\frac{1}{\rho_{e}C_{e}L_{s}}\left[\sigma_{I}\left(t\right)+\sigma_{R}\left(t\right)-\sigma_{T}\left(t\right)\right]$$

where $\sigma_{I}\left(t\right)$, $\sigma_{R}\left(t\right)$, and $\sigma_{T}\left(t\right)$ represent the incident, reflection, and transmission stresses, respectively, of the bar at time *t*; $A_{e}$, $\rho_{e}$, and $C_{e}$ represent the cross-sectional area, density, and p-wave velocity, respectively, of the elastic bar; and $A_{s}$ and $L_{s}$ represent the cross-sectional area and length of the sample, respectively.

**Figure 4:**
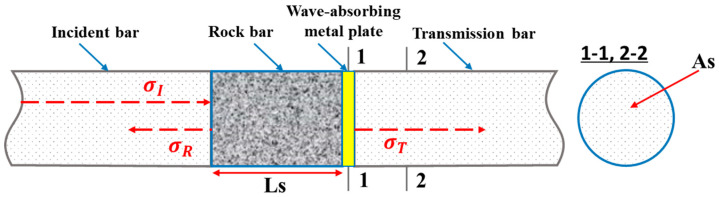
Stress-wave-propagation diagram.

The incident and reflected stress waves in the long, square granite bar can be measured using a strain gauge attached to it. The transmitted stress wave was measured using a strain gauge on the transmission bar, as shown in Figure 4. Therefore, the corresponding incident stress, reflected stress, and transmitted stress on the boundary test material can be obtained using the following formula [44,45]:

(4) $$\sigma_{I}=E_{1}\sigma_{I}\left(t\right)$$

(5) $$\sigma_{R}=E_{1}\sigma_{R}\left(t\right)$$

(6) $$\sigma_{T}=E_{2}\sigma_{T}\left(t\right)$$

where $E_{1}$ and $E_{2}$ are the elastic moduli of the granite and transmission bars, respectively. According to the SHPB test principle and the law of energy conservation, combined with Equations (4)–(6), the incident energy, reflection energy, and transmission energy on the boundary material can be calculated using the following formulas [46,47]:

(7) $$E_{I}=\frac{A_{e1}}{\rho_{e1}C_{e1}}\int_{0}^{\tau }\sigma_{I}^{2}\left(t\right)dt$$

(8) $$E_{R}=\frac{A_{e1}}{\rho_{e1}C_{e1}}\int_{0}^{\tau }\sigma_{R}^{2}\left(t\right)dt$$

(9) $$E_{T}=\frac{A_{e}}{\rho_{e}C_{e}}\int_{0}^{\tau }\sigma_{T}^{2}\left(t\right)dt$$

where $E_{I}$, $E_{R}$, and $E_{T}$ are the incident, reflection, and transmission energies of the boundary test material, respectively. τ denotes the duration of the stress wave. $A_{e1}$, $\rho_{\begin{array}{c} e1 \end{array}}$, and $C_{e1}$ represent the cross-sectional area, density, and P-wave velocity of the granite bar, respectively.

Since the end faces of the sample contacting the rod are coated with lubricant, the energy consumed by the friction at the end can be ignored in the calculation; therefore, the absorption energy of the boundary test material $E_{A}$ is [48,49,50]:

(10) $$E_{A}=E_{I}-E_{R}-E_{T}$$

### 3.2. Stress-Balance Check

**Figure 5:**
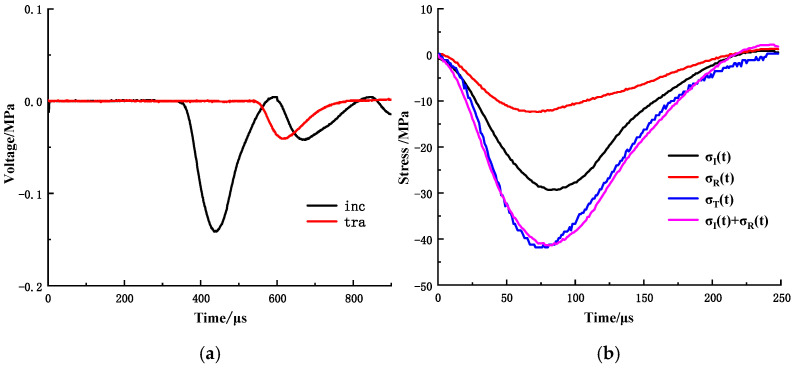
Stress-balance check of MP-A4-T2: (**a**) Waveform signal of MP-A4-T2; (**b**) Stress balance diagram of MP-A4-T2.

Balancing the dynamic stress during the impact-loading process is crucial for obtaining reliable test results. The spindle-shaped punch impinged on the incident bar and produced stress waves until the waves reached the dynamic stress balance after passing through the specimen three or four times. Before the experiment, the stress balance was checked. Waveform signals of the wave-absorbing metal-plate material MP-A4-T2 (aperture: 4 mm; thickness: 2 mm) recorded by the ultra-dynamic strain meter under static-dynamic coupling-loading conditions are shown in Figure 5a. According to formula (1)–(3), the stress-balance diagram of the boundary material under the condition of static-dynamic-coupling loading can be obtained, as shown in Figure 5b. It can be observed that the value of each point on the transmission-stress curve coincides with the sum of the incident and reflection stresses at the corresponding time, indicating that the dynamic stress-balance condition can be achieved and maintained in the dynamic loading process of the wave-absorbing metal plate, thus validating the test results.

After collating the data obtained from the super-dynamic strain gauge in the static-dynamic-coupling loading test of the wave-absorbing metal plates, the peak strength and energy dissipation characteristics of the metal plates with different pore sizes were calculated under the static-dynamic-coupling loading conditions. The changes in the test results of the wave-absorbing metal plates with different pore sizes are shown in Table 3.

### 3.3. Research on Wave-Absorbing Characteristics of Wave-Absorbing Metal Plates with Different Aperture Sizes

#### 3.3.1. Variation Law of Peak Stress and Reflectance under Different Aperture Sizes

(1) Effect of different aperture sizes on peak stress

**Figure 6:**
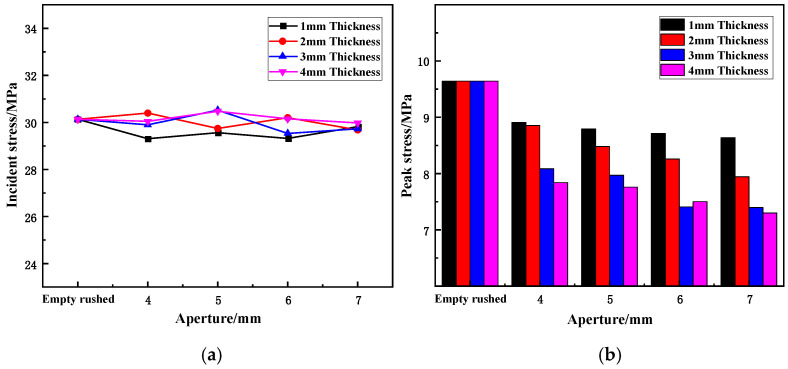
Variation trend of incident and reflected stress of wave-absorbing metal plate with increasing aperture size. (**a**) Variation in incident stress with aperture size. (**b**) Variation in peak stress with aperture size.

The peak stress of the sample is the dynamic strength of its dynamic stress–strain curve under static-dynamic-coupling loading, that is, the peak strength of the sample under a dynamic impact load, which reflects the impact resistance of the sample. Figure 6 shows the variation in the incident stress and peak stress of the wave-absorbing metal plate with the aperture under static-dynamic-coupling loading.

From Table 3 and Figure 6a, it can be observed that the incident stress in each impact test is relatively stable and fluctuates back and forth within a stable range without significant change. This indicates that although the rock bar was in a cyclic impact state, the impact load selected in the test was small. The rock bar is still in the elastic stage when stressed, and the degree of damage is weak; therefore, its influence on the test is also weak and can be ignored. At the same time, the stability of the incident stress shows that the conditions of the equal-energy shock are established, and the test results can be better analyzed in terms of the aperture size.

From the combined results of Table 3 and Figure 6b, it is evident that the aperture size has a certain influence on the reflected peak stress of the wave-absorbing metal plate. For the four different thicknesses, the peak stress of the wave-absorptive metal plate as a whole maintained a decreasing trend with an increase in the aperture size. When the wave-absorbing metal plate is fixed in place, the peak stress undergoes the largest reduction, with reduction rates of 7.62, 9.16, 16.1, and 18.7% for thicknesses of 1, 2, 3, and 4 mm, respectively. Among them, the metal plates with thicknesses of 1 and 2 mm exhibit the same reduction in peak stress, and the reduction for the 3 and 4 mm metal plates are similar to each other; the reduction in the peak stress decreases with an increase in the aperture. For samples with thicknesses of 1 mm and 2 mm, although the initial drop is identical and both are in a state of uniform drop, with the change in aperture size, the difference between the peak stresses of the two becomes increasingly larger. Moreover, the peak stress for the last two thicknesses becomes increasingly similar to the increase in the aperture size, finally reaching approximate coincidence.

(2) Effect of different aperture sizes on reflectivity variation

**Figure 7:**
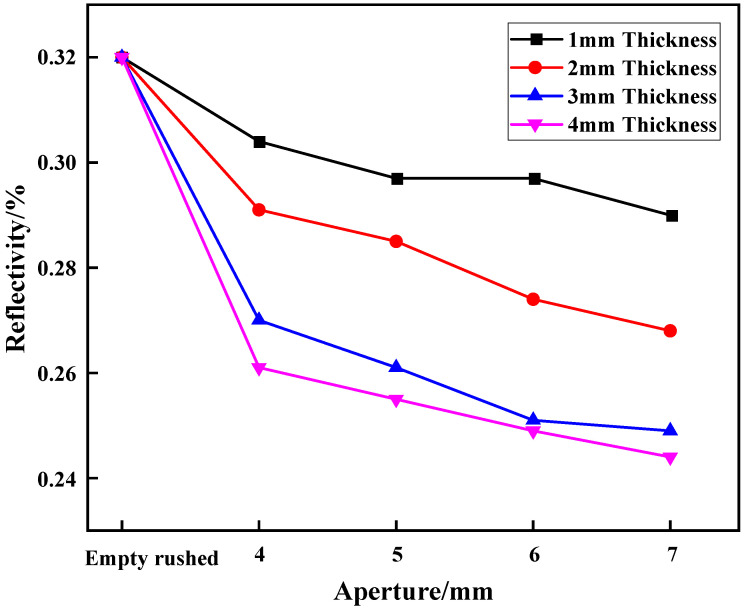
Variation trend of reflectivity of wave-absorbing metal plate with aperture size.

The reflectivity of the sample is the ratio of the reflected peak stress to the incident peak stress under static-dynamic-coupling loading, that is, the reflection intensity of the stress wave of the sample under the impact dynamic load, which reflects the degree of reduction in the wave-absorbing metal plate on the stress wave. Figure 7 shows the variation in reflectivity of the wave-absorbing metal plate with aperture size under static-dynamic-coupling loading.

By combining the results of Table 3 and Figure 7, it is apparent that the aperture size has a certain influence on the reflectivity of the wave-absorbing metal-plate sample. For the four different thicknesses, the reflectivity of the wave-absorptive metal plate as a whole maintained a decreasing trend with increasing aperture size. When the wave-absorbing metal plate was fixed in place, the reflectivity decreased the most, with drops of 5.06%, 9.06%, 15.63%, and 18.44% for aperture sizes of 1, 2, 3, and 4 mm, respectively. This variation trend is identical to that of the peak stress. This is due to the fact that, in general, the incident stress remains basically unchanged during the process of equal energy impact. According to the relationship among the three parameters, when the incident stress is constant, the peak-stress variation trend can be equivalent to the variation trend of reflectivity. Similarly, in this test, owing to the limitations of the granite bar itself, the incident stress will have a certain impact; therefore, the reflectivity can more accurately reflect the wave-absorbing effect of the wave-absorbing metal plate than the peak stress.

For the four different thicknesses, the reflectivity of the wave-absorptive metal plates decreases with increasing aperture size, which may be related to the ratio between the drilling area and the remaining area of the wave-absorptive metal plates. When the wave is transmitted to the metal plate, the compression wave and tensile wave generated by the drilling area and the remaining area on the boundary cancel each other near the boundary to achieve wave elimination. As the aperture size increases, the area of the borehole increases, the remaining area decreases, and the compression and tension waves generated by the boundary tend to be balanced, causing the reflectivity to decrease continuously.

#### 3.3.2. Variation Law of Energy Consumption of Wave-Absorbing Metal Plates with Different Aperture Sizes

(1) Effect of different aperture sizes on energy-absorption rate

**Figure 8:**
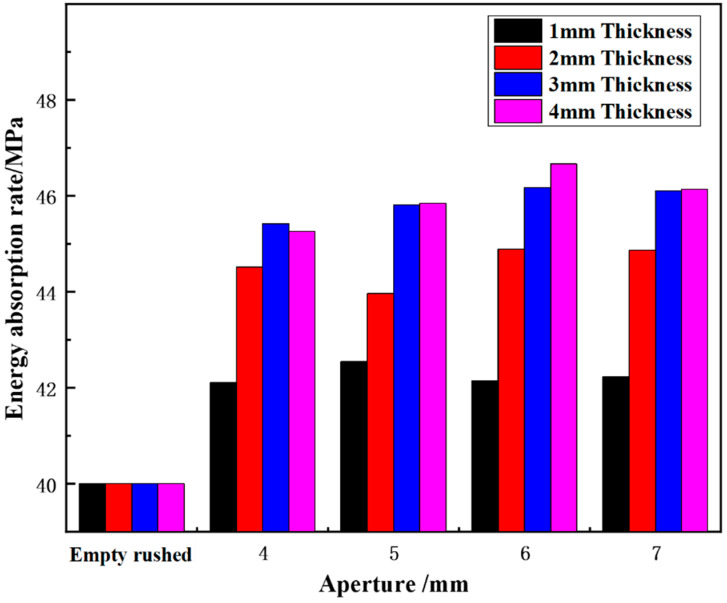
Variation trend of energy-absorption rate of wave-absorbing metal plate with aperture.

Figure 8 shows the variation in the energy-absorption rate of wave-absorbing metal-plate samples with different aperture sizes under static-dynamic-coupling loading. It can be seen from Figure 8 that for different aperture thicknesses, the energy-absorption rate of the wave-absorbing metal plates generally increases initially and then flattens out with the increase in aperture. When the aperture size was 6–7 mm, the energy absorption efficiency of the metal plate was the highest, and with increasing aperture size, the overall increase was also greater. When the aperture is in the range of 0–4 mm, the energy-absorption rate varies significantly, from 4.99% to 11.30%, 13.55%, and 13.15% depending on the thickness. However, when the aperture changed in the range of 4–6 mm, the overall energy-absorption rate of the sample increased slightly. When the aperture size ranged from 6–7 mm, the energy-absorption rate tended to be gradual and remained relatively unchanged. This is due to the fact that at the initial stage of aperture-size increase, the compression wave and tensile wave generated by the sample boundary do not achieve a balance. From the perspective of energy, this means that the ideal energy-absorption rate of the wave-absorbing metal plate is not reached at this time, and it is proportional to the aperture size before reaching the maximum energy-absorption rate. Therefore, with increased aperture size, the energy-absorption rate increased. However, when aperture size exceeds the critical value, the tensile wave generated on the sample surface becomes larger than the compression wave, and the energy-absorption rate also begins to decrease.

(2) Effect of aperture size on energy reflectance

**Figure 9:**
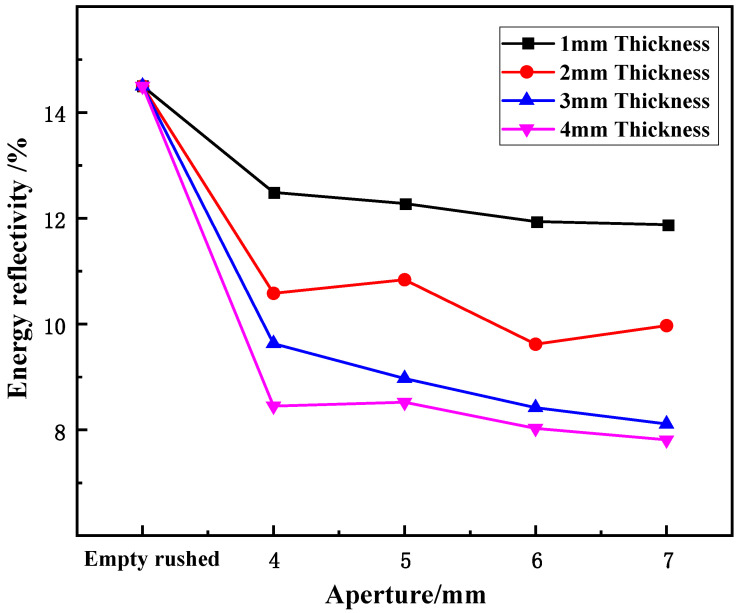
Variation trend of energy reflectance of wave-absorbing metal plate with aperture size.

Figure 9 shows the variation in energy reflectance with axial compression of samples containing cracks under static-dynamic-coupling loading. It can be seen in Figure 9 that for different thicknesses, the variation law of the wave-absorbing metal-plate samples with increasing aperture is opposite to the energy-absorption rate; that is, the energy reflectance of the samples as a whole decreases slightly initially and then flattens out with the increase in aperture size. The energy reflectance of the metal plate was a minimum when the aperture size was 6–7 mm, and the overall decrease was larger with increasing aperture size. When the aperture size changes in the range of 0–4 mm, that is, when the wave-absorbing metal plate is added, the energy reflectance of the sample changes significantly, by 14.26%, 27.18%, 33.59%, and 41.7% for thicknesses of 1, 2, 3, and 4 mm, respectively. When the aperture size changed in the range of 4–6 mm, the energy reflectance of the sample decreased slightly overall, and when the aperture size changed in the range of 6–7 mm, the energy reflectance tended to be flat, and the variation range was relatively insignificant. The reason for this is that at the initial stage of the aperture increase, the compression wave and tensile wave generated by the sample boundary do not reach a balance. From an energy perspective, the maximum reduction in energy by the wave-absorbing metal plate has not been achieved at this time, and it is inversely proportional to the aperture size before reaching the minimum energy reflectivity. Therefore, as the aperture size increased, the energy reflectance rate decreased. However, the tensile wave generated on the sample surface grows larger than the compression wave, and the energy reflectance also begins to increase.

(3) Effect of aperture on energy transmittance

**Figure 10:**
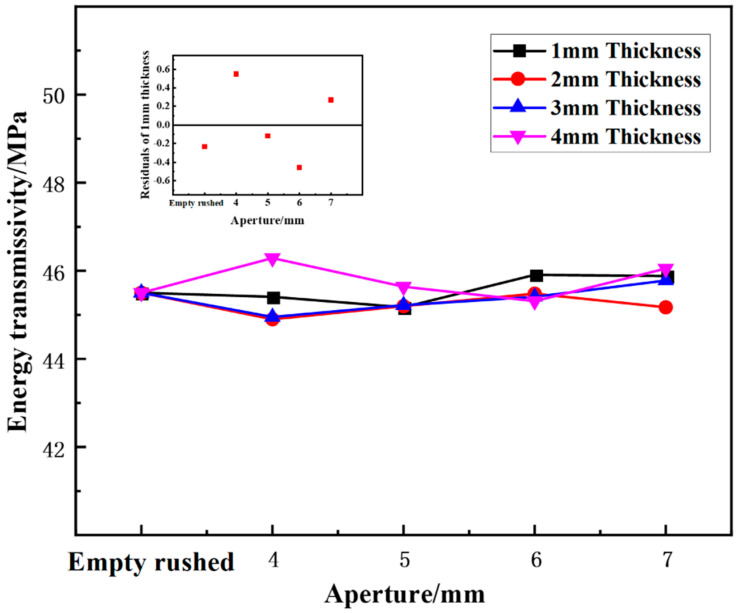
Variation trend of energy transmittance of wave-absorbing metal plate with aperture.

Figure 10 shows the variation in the energy transmittance of the wave-absorbing metal plate samples with aperture size under static-dynamic-coupling loading. By combining the results of Table 3 and Figure 10, it can be seen that under different thicknesses, the energy transmittance of the wave-absorbing metal-plate samples initially decreases and then increases with the increase in aperture size, but the overall variation range is small relative to the energy-absorption rate and energy reflectance. At thicknesses of 1 and 2 mm, the energy transmittance tends stabilize with an increase in aperture.

### 3.4. Study on Wave-Absorbing Characteristics of Wave-Absorbing Metal Plate with Different Thicknesses

#### 3.4.1. Variation Law of Peak Stress and Reflectance under Different Thicknesses

(1) Effect of thickness on peak stress and reflectivity

**Figure 11:**
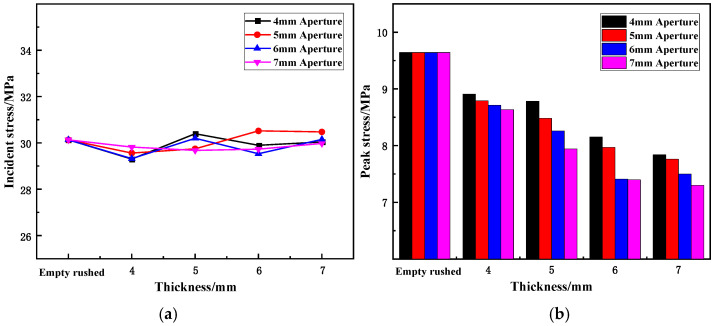
Variation trend of incident and reflected stresses of wave-absorbing metal plate with thickness. (**a**) Variation in incident stress with thickness; (**b**) Variation trend of peak stress with thickness.

Based on the test results in Table 3, the variation trend of the incident and peak stresses of the wave-absorbing metal-plate sample with increasing thickness under static-dynamic-coupling loading are illustrated in Figure 11. From Table 3 and Figure 11a, it is apparent that the incident stress in each impact test is relatively stable, as it fluctuates back and forth within a given interval without any major changes. This behavior indicates that although the granite bar is in a cyclic impact state, the impact load selected for the test is small; therefore, the granite bar is still in the elastic stage when it is stressed, and the damage degree is weak; as a result, the impact on the test is also negligible and can be ignored. At the same time, the stability of the incident stress also demonstrates that the conditions of the test equal-energy impact can be established, and the test results can be better analyzed in terms of metal-plate thickness.

Table 3 and Figure 11b together indicate that the thickness has a certain influence on the reflected peak stress of the wave-absorbing metal plate. The peak stress of the wave-absorbing metal plate decreased with increasing thickness of the metal plate from 4 to 7 mm. When the wave-absorbing metal plate was fixed in place, the peak stress decreased the most, with decreases of 7.61, 8.82, 9.62, and 10.42% for thicknesses of 4, 5, 6, and 7 mm, respectively. In the process of increasing the thickness of the wave-absorbing metal plate, the peak stress decreases with increasing thickness. The peak stress of the metal-plate samples with 4, 5, 6, and 7 mm apertures decreased by 11.00%, 11.72%, 27.38%, 13.93%, and 15.47%, respectively, for a plate thickness of 4 mm compared with that of 1 mm. Although the peak stress decreases with an increase in the metal-plate thickness, the peak stress of the sample with different apertures is still affected by the plate thickness, and the difference increases with an increase in the aperture.

**Figure 12:**
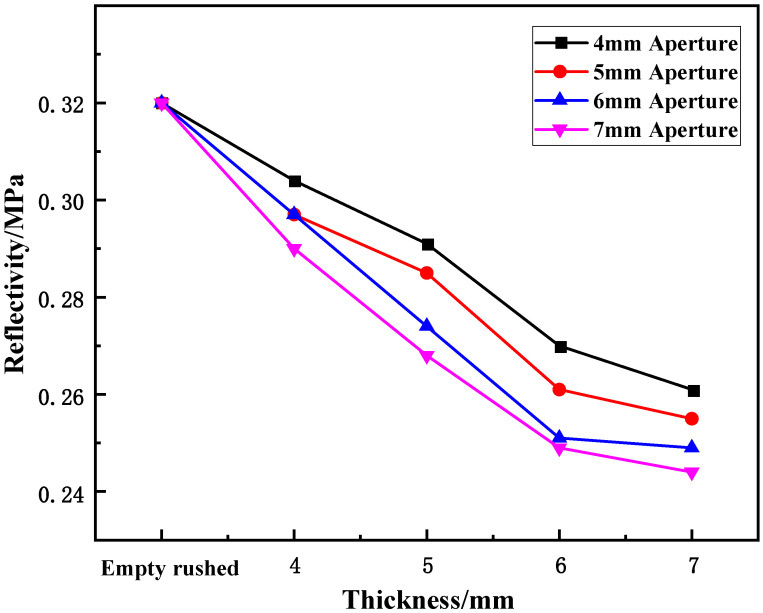
Variation trend of reflectivity with thickness of wave-absorbing metal plate.

Combining the results of Table 3 and Figure 12, it can be seen that the thickness has a significant influence on the reflectivity of the wave-absorbing metal-plate sample. The reflectance of the wave-absorbing metal plates decreases with increasing thickness from 4 to 7mm. When the wave-absorbing metal plate was fixed in place, the reflectivity decreased the most, with drops of 5.01%, 7.06%, 7.12%, and 9.50% for thicknesses of 4, 5, 6, and 7 mm, respectively. When the thickness of the sample was in the range of 1–3 mm, the reflectance of the sample decreased with increasing thickness. When the sample was in the thickness range of 3–4 mm, its reflectance showed a decreasing trend, but the decreasing range was relatively small. Although the variation in reflectivity is similar to that of the peak stress, the incident stress remains unchanged during the process of equal-energy impact. According to the relationship among the three parameters, when the incident stress is constant, the variation trend of the peak stress can be equal to the variation trend of reflectivity. Similarly, in this test, owing to the limitations of the granite bar itself, the incident stress will be affected to a certain extent; therefore, the reflectivity can more accurately reflect the wave-absorbing effect of the wave-absorbing metal plate than the peak stress.

For four different thicknesses, the reflectivity of the wave-absorptive metal plate decreased with increasing thickness. The decreasing trend starts to plateau when the thickness is 3 mm, indicating that the thickness has a critical value for the reduction in reflectivity. Before this value, a linear relationship exists between thickness and reflectivity, and beyond this value, increasing the thickness of the metal plate had no effect on reflectivity.

#### 3.4.2. Energy Consumption Variation in Wave-Absorbing Metal Plate with Different Thicknesses

(1) Effect of thickness on energy absorption

**Figure 13:**
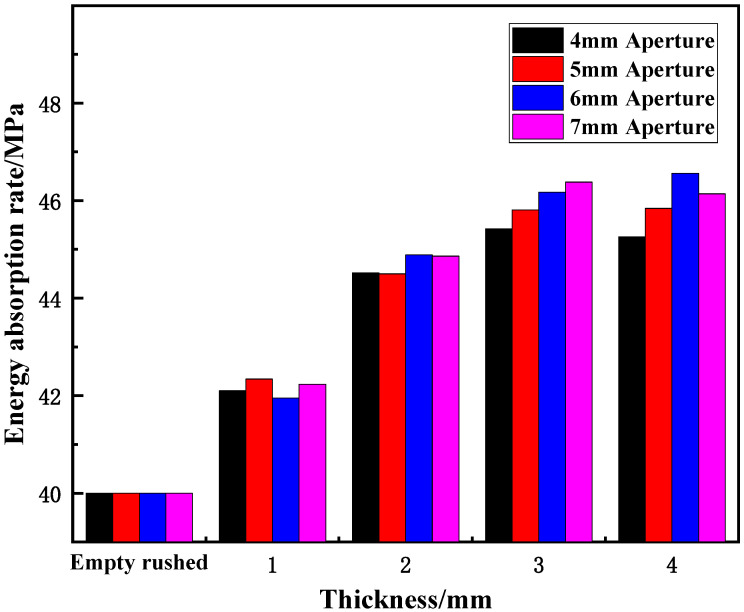
Variation trend of energy-absorption rate of wave-absorbing metal plate sample with thickness.

Figure 13 shows the variation in the energy-absorption rate of cracked specimens with thickness under static-dynamic-coupling loading. It can be seen from Figure 13 that the energy-absorption rate of the wave-absorbing metal-plate samples increases with increasing thickness only when the thickness is changed, and the increase in the energy-absorption rate decreases with an increase in thickness. When the thickness of the wave-absorbing metal plate was varied from 0 to 2 mm, the energy-absorption rate of the sample increased significantly; in the thickness range of 2–3 mm, the rate of increase in energy absorption decreases compared with that described in the previous section; and in the range of 3–4 mm, the increase in the energy-absorption rate tended to be stable. This is case as in the initial stage of the thickness change, the thickness of the sample affects its compressible region after being impacted. Within a certain range of thickness, the compressible range of the sample is directly proportional to the thickness after being impacted by equal energy; however, beyond this range, simply increasing the thickness will not improve the energy absorption efficiency of the sample. As shown in Figure 13, this critical value was approximately 3 mm.

(2) Effect of thickness on energy reflectance

**Figure 14:**
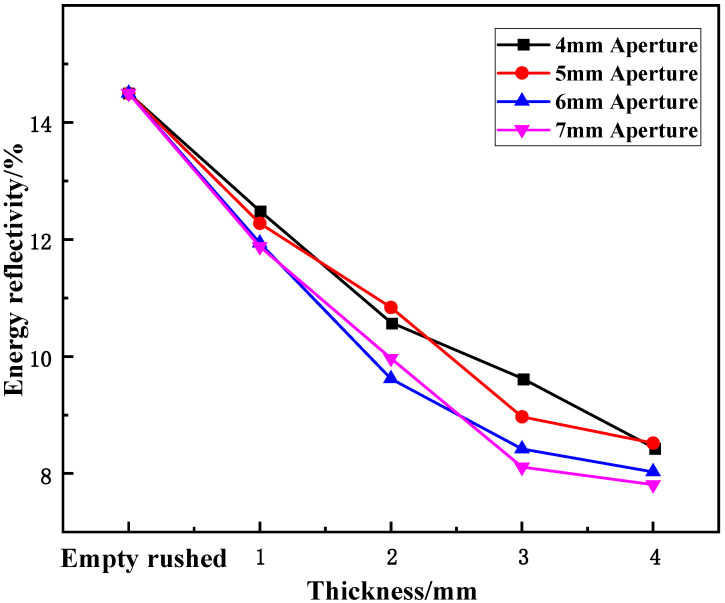
Variation trend of energy reflectance of wave-absorbing metal plate sample with thickness.

Figure 14 shows the variation in energy reflectance with the thickness of the cracked samples under static-dynamic-coupling loading. From Table 3 and Figure 14, it can be seen that the variation in the wave-absorbing metal-plate sample with thickness is opposite to that of the energy-absorption rate at each aperture. In other words, the overall energy reflectance of the sample first decreases and then tends to stabilize with increasing plate thickness, where the greater the thickness, the smaller the overall decrease: for plate thicknesses in the range of 0 to 2 mm, the energy reflectance of the sample decreases, and for thicknesses of 2–3 mm, the decreasing range of energy reflectance begins to decrease compared with the previous period. However, when the thickness is in the range of 3–4 mm, the decrease in energy reflectance tends to be stable. This stability is due to the initial stage of the thickness change, in which the thickness of the sample affects its compressible region after impact. Within a certain thickness range, the compression range of the sample is proportional to the thickness after being impacted by equal energy. However, beyond this range, simply increasing the thickness of the plate does not improve the energy absorption efficiency. As shown in Figure 14, this critical value was approximately 3 mm.

(3) Effect of thickness on energy transmittance

**Figure 15:**
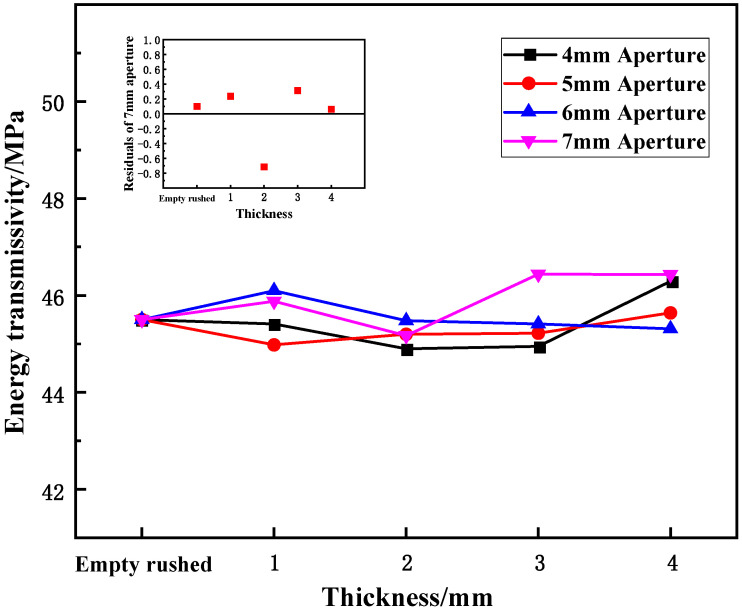
Variation trend of energy transmittance of wave-absorbing metal plate samples with thickness.

Figure 15 shows the variation in the energy transmittance of cracked specimens with axial compression under static-dynamic-coupling loading. Combined with the results of Table 3 and Figure 15, it is apparent that the energy transmittance of the wave-absorbing metal-plate samples fluctuates back and forth with increasing thickness, but the overall range of change is not obvious compared to that of the energy-absorption rate and energy reflectance. For 4 and 5 mm aperture sizes, the energy transmittance tends to stabilize with increasing thickness.

## 4. Conclusions

In this study, the effects of thickness and aperture size on the energy-absorption characteristics of wave-absorbing metal plates under static-dynamic-coupling loading were studied. The characteristics of the incident- and reflection-stress curves, reflectance, and energy consumption of the wave-absorbing metal-plate samples were analyzed, and the following conclusions were drawn.

(1) The aperture size and thickness of the MP wave-absorbing metal plate significantly affect the peak stress and reflectance of the samples under static-dynamic-coupling loading conditions. The peak stress and reflectance of the samples first increased and then decreased with increasing aperture size, and the peak stress decreased the most when the metal plate was fixed in place. However, the peak stress and reflectivity decrease with increasing aperture size. The variation trend of the peak stress and reflectance with thickness is similar to that of the aperture, but the thickness has a greater effect.
(2) The energy-absorption rate of the wave-absorbing metal-plate samples increases with an increase in aperture size and thickness under static-dynamic-coupling loading conditions. The energy-absorption efficiency increases the most when the wave-absorbing metal plate is fixed in place, and the increase in the energy absorption efficiency tends to be gradual with increasing aperture size and thickness. The energy-absorption efficiency is at a maximum when the aperture size is 6–7 mm and the metal-plate thickness is 3–4 mm. By further increasing the pore size and thickness, the energy-absorption rate exhibited a downward trend. The variation trend of the energy reflectance of the wave-absorbing metal plate is opposite to that of the energy absorption, and the energy reflectance decreases with increasing aperture size and thickness. The decrease in energy reflectance is the largest when the metal plate is initially fixed in place, whereas the decrease in energy reflectance tends to be gradual with the increase in aperture size and thickness. Similarly, the energy reflectance reaches its minimum value in the aperture-size range of 6–7 mm and metal-plate-thickness range of 3–4 mm. The energy transmittance of the wave-absorbing metal plate fluctuates within a stable range, and the variation range is not obvious compared with that of the energy-absorption rate and reflectance.
(3) The boundary material wave-absorbing metal plate selected in this paper is placed between the rock and the transmission end, which can achieve the effect of eliminating the transmitted wave, reflection and energy absorption, and can meet the test requirements under the condition of true-triaxial strong disturbance.

## Figures and Tables

**Table 1:** Material parameters of wave-absorbing metal plate.

Sample Number	Diameter/(mm)	Aperture/(mm)	Thickness/(mm)	Hole Margin/(mm)
MP-A4-T1	50	4.0	1.0	3.0
MP-A5-T1	50	5.0	1.0	3.0
MP-A6-T1	50	6.0	1.0	3.0
MP-A7-T1	50	7.0	1.0	3.0
MP-A4-T2	50	4.0	2.0	3.0
MP-A5-T2	50	5.0	2.0	3.0
MP-A6-T2	50	6.0	2.0	3.0
MP-A7-T2	50	7.0	2.0	3.0
MP-A4-T3	50	4.0	3.0	3.0
MP-A5-T3	50	5.0	3.0	3.0
MP-A6-T3	50	6.0	3.0	3.0
MP-A7-T3	50	7.0	3.0	3.0
MP-A4-T4	50	4.0	4.0	3.0
MP-A5-T4	50	5.0	4.0	3.0
MP-A6-T4	50	6.0	4.0	3.0
MP-A7-T4	50	7.0	4.0	3.0

**Table 2:** Test equipment parameters.

Test Equipment	Material	Length/ (mm)	Diameter/ mm	Density/ $kg\cdot m^{-3}$	Wave Velocity/ $\left(m\cdot s^{-1}\right)$	Wave Impedance/$\left(g\cdot cm^{-3}\right)\cdot \left(m\cdot s^{-1}\right)$
Incident bar	40Cr Alloy steel	1500	50	7800	5400	42,120
Transmission bar	40Cr Alloy steel	1500	50	7800	5400	42,120
Rock bar	Granite	1000	50	2547	4300	10,952

**Table 3:** Test results of wave-absorbing metal-plate materials.

Sample Number	Incidence Peak Intensity/MPa	Reflection Peak Intensity/MPa	Reflectivity	Total Incident Energy/J	Total Reflected Energy/J	Total Transmitted Energy/J	Total Absorbed Energy/J	Energy Reflectance	Energy Transmittance	Energy Absorption Rate
MP-A4-T1	29.30	8.91	0.304	12.15	1.52	5.52	5.12	0.125	0.454	0.421
MP-A5-T1	29.56	8.79	0.297	12.08	1.48	5.46	5.14	0.123	0.452	0.425
MP-A6-T1	29.32	8.71	0.297	12.38	1.48	5.69	5.22	0.119	0.459	0.421
MP-A7-T1	29.82	8.64	0.290	12.13	1.44	5.56	5.12	0.119	0.459	0.422
MP-A4-T2	30.40	8.86	0.291	12.68	1.34	5.69	5.65	0.106	0.449	0.445
MP-A5-T2	29.74	8.48	0.285	12.10	1.31	5.47	5.32	0.108	0.452	0.440
MP-A6-T2	30.20	8.26	0.274	12.65	1.22	5.76	5.68	0.096	0.455	0.449
MP-A7-T2	29.68	7.94	0.268	11.55	1.15	5.22	5.18	0.100	0.452	0.449
MP-A4-T3	29.90	8.09	0.270	11.38	1.10	5.12	5.17	0.096	0.449	0.454
MP-A5-T3	30.52	7.97	0.261	13.27	1.19	6.00	6.08	0.090	0.452	0.458
MP-A6-T3	29.53	7.41	0.251	12.14	1.02	5.51	5.60	0.084	0.454	0.462
MP-A7-T3	29.73	7.40	0.249	12.44	1.01	5.70	5.74	0.081	0.458	0.461
MP-A4-T4	30.04	7.84	0.261	12.44	1.05	5.76	5.63	0.084	0.463	0.453
MP-A5-T4	30.47	7.76	0.255	12.61	1.07	5.76	5.78	0.085	0.456	0.458
MP-A6-T4	30.15	7.50	0.249	12.77	1.03	5.79	5.96	0.080	0.453	0.467
MP-A7-T4	29.98	7.30	0.244	12.50	0.98	5.76	5.77	0.078	0.460	0.461
